# The Pathogenesis of Nontraumatic Osteonecrosis

**DOI:** 10.1155/2012/601763

**Published:** 2012-11-08

**Authors:** Jesse Seamon, Thomas Keller, Jamal Saleh, Quanjun Cui

**Affiliations:** Department of Orthopaedic Surgery, University of Virginia School of Medicine, 400 Ray C. Hunt Drive, Box 800159, Charlottesville, VA 22908, USA

## Abstract

Nontraumatic osteonecrosis continues to be a challenging problem causing debilitating major joint diseases. The etiology is multifactorial, but steroid- and alcohol-induced osteonecrosis contribute to more than two thirds of all cases with genetic risk factors playing an important role in many other cases, especially when they contribute to hypercoagulable states. While the exact mechanisms remain elusive, many new insights have emerged from research in the last decade that have given us a clearer picture of the pathogenesis of nontraumatic osteonecrosis of the femoral head. Progression to end stage osteonecrosis of the femoral head appears to be related to four main factors: interactions involving the differentiation pathway of osteoprogenitor cells that promote adipogenesis, decreased angiogenesis, direct suppression of osteogenic gene expression and proliferation of bone marrow stem cells, and genetic anomalies or other diseases that promote hypercoagulable states.

## 1. Introduction

Nontraumatic osteonecrosis (ON) of the femoral head continues to represent a significant challenge to orthopaedic surgeons [1–3] and is a devastating disease for affected patients with complete collapse of the femoral head occurring in 80% of untreated patients [4, 5]. Nontraumatic conditions associated with ON are numerous [1, 2, 6, 7]. Some of the well accepted and common associations include corticosteroid use [8, 9], alcohol abuse [10], systemic lupus erythematous [11], hemoglobinopathies including sickle cell anemia [12–14], Legg-Calve Perthes disease [15], and exposure to radiation [16] or cytotoxic agents [17]. Other less common associations include Gaucher's disease [11, 18, 19], dysbarisms [4], HIV [20], hyperlipidemia [21], pancreatitis, and gout [22]. A substantial number of cases have no identifiable etiologic factors and have been referred as idiopathic [23]. The pathogenic mechanisms leading to impaired circulation in these conditions are most likely multifactorial [3, 21, 24, 25]. Mechanical blood vessel interruption, thrombotic intravascular occlusion, and extravascular compression are the three most commonly accepted general mechanisms leading to ON [1, 3, 24, 26]. Recent attention has been given to the interplay between individual genetic predisposition and environmental factors related to ON [26–50]. Both heritable and acquired risk factors for femoral head ON related to hypercoagulability [28, 32–36, 47, 50–52], hemoglobinopathies [27, 28, 36], steroids [48], angiogenesis [43], and oxidative stress [45] have been identified in many patients. Technological advances in molecular biology have enabled studies on the advanced mechanisms of steroid and alcohol-induced ON [9, 10, 53–73]. Preliminary data with statins and low molecular weight heparin from clinical studies are promising (Table 1) [74, 75]. Still, the exact pathogenesis of ON is controversial and poorly understood. This limited knowledge has impeded the development of any effective prophylaxis or pharmacologic treatment for this debilitating disease [1, 6]. Currently, the primary effective treatments are surgical, consisting of unloading osteotomies, vascularized grafts, and total hip replacements [4, 5, 21, 26]. This report seeks to systematically review the more common causes of pathogenesis of nontraumatic ON of the femoral head, which include steroids, alcohol, genetic factors, and hypercoagulability.

## 2. Pathophysiology of Osteonecrosis

**Figure 1:**
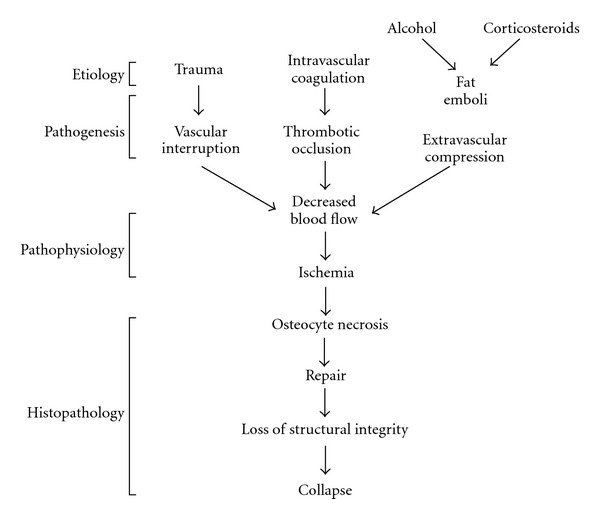
Schematic representation of the development of osteonecrosis. (Reprinted, with permission, from [24].)

Ultimately, ON of the femoral head occurs through one final common pathway, which is decreased blood flow to the femoral head that leads to bone ischemia and death [1, 3, 21, 24] (Figure 1). However, the precipitating mechanisms which lead to this final pathway are varied. Vascular occlusion can be caused by local thrombi, fat emboli, nitrogen bubbles, or abnormally shaped red blood cells [52, 76–78]. Extravasated blood along with fat or cellular elements in the marrow cavity can extrinsically compress both arteries and veins [7, 10, 54, 79, 80]. The blood vessels in the femoral head may be directly damaged by vasculitis, irradiation, or chemical toxicity [1, 2, 7]. Bone cells may remain viable if the collateral circulation is sufficient. Although bone has a relatively rich vascular supply, the distribution is inhomogeneous and leaves some areas more vulnerable than others [7, 81]. Once the ischemic threshold is reached, the morphologic changes within the affected bone are similar regardless of the inciting disorder.

No histologic evidence of damage is evident until twenty-four to seventy-two hours after vascular compromise [81]. Examination of the bone marrow reveals necrosis of hematopoietic cells, endothelial cells, and lipocytes. Osteocytes atrophy and die, and increasing numbers of empty lacunae become evident with time. The release of lysosomes acidifies the surrounding tissue as dying lypocytes release free fatty acids which saponify with extracellular calcium to form insoluble soaps. A subsequent increase in fatty marrow water content is detectable by magnetic resonance imaging (MRI) and represents the earliest abnormality seen clinically.Saponified fats and other necrotic areas eventually calcify and can be detected by plain radiographs later in the disease process [81–83].

Cell death is followed by initiation of the repair process. An inflammatory cascade is initiated by the adjacent viable tissues, which leads to a fibrous vascular in growth in the regions of cell death. Vascular canals can be seen penetrating the medullary canals of the cancellous bone and the haversian canals in the overlying cortical bone. These vessels are accompanied by primitive mesenchymal cells which differentiate into osteoblasts and osteoclasts [81–84]. Immature woven bone is deposited throughout the network of dead trabecular bone. The nonviable trabecular bone is slowly resorbed by the process of creeping substitution. Unfortunately, the newly deposited bone does not attain the previous structural integrity of that region of the femoral head, leading to subchondral collapse of these regions at appropriate weight bearing loads. Ultimately, this leads to irregularities in the normally smooth cartilaginous surface of the femoral head that progress to end-stage arthritis. Additionally, surrounding viable bone may lose mass and become osteopenic from relative patient inactivity over the course of the disease [1, 25, 81–84]. 

## 3. Historical Views on the Pathogenesis of Osteonecrosis

Phemister was the first to propose that the “aseptic necrosis” might result from fractures, bone graft transplantation, radiation and vascular obstruction from thrombosis, or embolization [85]. ON was later regarded as a primitive vascular problem. Some believed that ON of the femoral head resulted from a type of vasculitis [86]. Chandler implicated an extraosseous embolic process and introduced the concept of “coronary disease of the hip” [87]. In a microangiographical study of 31 femoral heads with idiopathic ON, Atsumi found extraossesous interruption of the superior retinacular arteries along with early angiogenesis and compensatory hypertrophy of the unaffected surrounding vasculature. They also found blockage of revascularization occurring along the areas of subchondral collapse in the weight-bearing region [88]. In contrast, Glimcher and Kenzora found no evidence to support blood vessel involvement in 150 adult femoral heads with ON and instead implicated a purely metabolic syndrome leading to cell death [82–84]. 

 Many theories regarding the pathogenesis of nontraumatic ON have been proposed over the past twenty years [1, 6, 21, 24]. Intraosseous hypertension, intravascular fat or gaseous emboli, and extravascular compression by increased marrow fat stores are several accepted theories [7, 52, 54, 78–80, 89–91]. Most support a “multiple hit” theory, with accumulated tissue stress from various insults reaching a critical threshold and initiating the disease process [1, 25]. Many would agree that these theories are not mutually exclusive, but are instead mutually supportive. 

## 4. Association of Osteonecrosis with Hypercoagulopathy and Genetic Alterations

Paul-Jones first suggested that hypercoagulability, and in specific intravascular coagulation could be a cause of ON in 1992 [77]. Thrombotic occlusion of the microcirculation can occur from hereditary thrombophilia, impaired fibrinolysis or antiphospholipid antibodies [16]. Additional causes include environmental or acquired/preexisting conditions, such as hyperlipidemia, hypersensitivity reactions, thromboplastin release during pregnancy, malignant tumors, and inflammatory bowel disease all may contribute additional risk to individuals with an underlying genetic predisposition to form microvascular thrombi [16, 21, 26, 29, 32, 34, 50, 72, 73, 75, 78, 92–94]. The role of sickle cell disease and other hemoglobinopathies in promoting ON of the femoral head has been well documented and also seems to act through the final pathway of intravascular coagulation [12–14, 95–98].

Björkman et al. showed in a retrospective study of 63 adult patients with osteonecrosis of the femoral head, that mutations in the factor V Leiden or the prothrombin 20210A gene were significantly more frequent in patients with idiopathic osteonecrosis than in patients with steroid or alcohol-induced osteonecrosis, as well as in a population of healthy control subjects [99]. This is supported by Zalavras and his colleagues who have demonstrated that the factor V Leiden mutation was presented in 18% of 72 adult Caucasian patients, compared with 4.6% of 300 healthy control subjects [50]. In addition, protein C and protein S deficiencies, which lead to thrombophilia have been associated with ON of the femoral head [32]. Jones et al. have studied the blood samples of 45 patients with osteonecrosis, 5 of which had no known preexisting conditions that could cause ON otherwise. In comparison to 40 age matched healthy controls, patients with ON were 3 times more likely to have a gene abnormality promoting anticoagulation, and further in those with no preexisting condition 100% had a gene abnormality promoting anticoagulation [40]. Glueck et al. have done extensive work in profiling the role of hypercoagulability and ON [30, 33–35, 72, 75, 93]. In a population of 36 patients entering a treatment trial with low-molecular-weight heparin (LMWH) for treatment of femoral head osteonecrosis they found gene polymorphisms that resulted in increased activity of the plasminogen activator inhibitor-1 gene (PAI-1), alterations in the methylenetetrahydrofolate reductase (MTHFR), and resultant hypofibrinolysis concurrent with higher homecysteine and lipoprotein levels than controls [34]. In this same group of patients, they showed that enoxaparin prevented progression of osteonecrosis in patients with early stage disease [75]. Chang et al. found that polymorphism in the MTHFR gene increased the risk for ON in the Korean population [28], while Kim et al. were unable to show a significant association between single nucleotide polymorphisms (SNPs) in the MTHFR gene locus and development of ON in the Korean population [42].

It has become clear that in general, genetic anomalies or heritable diseases that promote intravascular coagulation are associated with the development of osteonecrosis. Future studies will likely focus on ways to screen for and localize polymorphisms associated with hypercoagulability, so that earlier or perhaps even prophylactic treatment can be provided for this patient population.

### 4.1. Genetic Associations

As previously discussed, thrombophilic disorders caused by both heritable diseases and genetic anomalies such as SNPs play a major role in the etiology and progression of ON due to genetic alterations in key components in the coagulation cascade including Protein C, Protein S, PAI-1, and a number of other factors [12–14, 16, 27, 30, 33–35, 72, 78, 93, 95–98]. Chen et al. evaluated two Taiwanese pedigrees with familial autosomal dominant osteonecrosis of the femoral head. They were able to link the presence of mutations in the Protein C, Protein S, and PAI-1 proteins to the 2q13-q14, 3q11.1-q11.2 and 7q21.3-q22 chromosomal segments respectively [30]. Pierre-Jacques et al. have reported on a familial heterozygous Protein S deficiency in a patient with multifocal osteonecrosis [49].

Numerous other genetic associations have been identified [27, 28, 31, 33, 35–38, 43–48]. Glueck et al. demonstrated that the T-786C Drosophila nitric-oxide synthase (dNOS) SNP results in decreased activity of nitric oxide which is responsible for promoting angiogenesis, bone formation, and inhibiting platelet aggregation. 22% of patients with idiopathic ON in their series had this SNP compared to only 5% of controls [33, 35]. Similarly, Koo et al. have found that polymorphisms in the nitric oxide synthase gene increased the risk of ON in their study population [47]. Hong et al. evaluated SNP in the transferrin (TF), vascular endothelial growth factor C (VEGFC), sterol regulatory element binding transcription protein-3 (IGFBP3), and angiotensin I converting enzyme (ACE) genes in a comparison study between 450 patients with femoral head ON and 300 matched healthy controls. They found that the SNP R2453839S, SNP on the IGFBP3 gene was significantly associated with development of ON and SNPs in the ACE gene were associated with increased chance of progression of steroid induced ON. Surprisingly, they found that SNPs in thekinase insert domain receptor (KDR) and neuropilin 1 (NRP1) gene loci were associated with decreased prevalence of ON [38]. Kim et al. have done extensive work in the Korean population to identify possible genes where SNPs may be related to increased development on ON [38, 41, 43–46]. They have identified SNPs in the SREBP-2 gene [41], interleukin receptor 23 gene [44], annexin gene family [46], catalase gene [45], and promoter polymorphisms in the VEGF gene [43]. Dai et al. have shown that polymorphisms in genes that inhibit the tissue factor pathway may lead to increased risk of osteonecrosis [31]. Beyond the previously discussed genes, polymorphisms invitamin D receptor (VDR) gene, the thymidylate synthase gene (TYMS), and the Type II collagen A1 (COL2A1) gene have all been identified as increasing the risk of ON of the femoral head [36]. 

Not all patients receiving high-dose steroids develop ON. Asano et al. have postulated that differences in drug metabolism related to genetic variation may explain why some develop ON while others do not [73, 92]. They examined 136 patients after a kidney transplant and found a strong association between those expressing a specific nucleotide polymorphism in the gene encoding the transport protein P-glycoprotein (P-gp) and resistance to ON. P-gp plays an important role in the absorption and distribution of drugs. By measuring serum through levels of tacrolimus, an immunosuppressive drug whose bioavailability is known to be influenced by P-gp, they were able to correlate enhanced P-gp activity with resistance to ON. The increased P-gp activity may lead to more rapid steroid clearance and subsequently lower serum steroid concentrations. Furthermore, they found that individuals expressing the C3435TT genotype of the gene encoding P-gp had significantly higher P-gp activity and a significantly lower incidence of ON. Their results demonstrate a genetically linked resistance to steroid-induced ON in patients with the C3435TT genotype of the gene encoding P-gp [73, 92]. He and Li have shown that the P-glycoprotein gene ABCB1 which modulates glucocorticoid uptake may be associated with development of ON [37]. In a comparison of patients on chronic glucocorticoids with and without ON they found that the G2677T/A SNP was associated with development of steroid induced ON [37]. Kim et al. also found SNPs in the ABCB1 (c3435t) gene to be related to increased sensitivity to steroid induced ON and found that concomitant SNPs in the CBP (r3751845s) gene increased the relative sensitivity [45].

Chao et al. proposed that genetic polymorphisms may influence the occurrence of alcohol-induced osteonecrosis [29]. Polymorphisms of several alcohol-metabolizing enzymes were evaluated in alcoholic patients with hip osteonecrosis, pancreatitis, and cirrhosis of the liver. They found that allele frequencies of ADH2*1 (alcohol dehydrogenase) and ALDH2*2(aldehyde dehydrogenase) differed among these disease-defined subpopulations of alcoholics, suggesting that differences in the alcohol metabolizing enzyme genes may be responsible for different organ-specific complications [29]. 

As our knowledge about to the genetic basis of ON improves, so too will our ability to identify at-risk individuals. Certainly, genetic polymorphisms in the genes that help us to metabolize alcohol and steroids along with alterations in the genetic make-up of genes involved in the coagulation cascade seem to be the main candidates for increasing risk to non-traumatic femoral head osteonecrosis.

## 5. Steroid and Alcohol-Induced Osteonecrosis

Exogenous corticosteroids and alcohol have all been associated with nontraumatic osteonecrosis. The dose affects of these substances and exact mechanism by which they produce osteonecrosis remains unknown though currently a topic of investigation.

### 5.1. Steroids

The association between excessive corticosteroid use and the development of ON has been well established since the first case report in a patient with rheumatoid arthritis in 1957 [100]. The incidence of ON has increased in parallel with the rise in glucocorticoid therapy for treatment of systemic conditions as well as organ transplantation. Of the 30 million Americans currently receiving glucocorticoid therapy, as many as 40% will develop some degree of osteonecrosis [70, 101]. Glucocorticoids are the most common cause of nontraumatic osteonecrosis [23]. Patients receiving steroid therapy have an approximately 20-fold increase in their likelihood of developing ON [8]. Though the dose effect of corticosteroid therapy on osteonecrosis remains largely unknown, recent studies suggest that corticosteroid doses above 25–40 mg/day are significant risk factors for nontraumatic ON in renal transplant and SLE patients [94, 102]. Several hypotheses regarding the cause of steroid-induced ON are based around the notions of small vessel occlusion by fatty emboli and the impedance of sinusoidal blood flow secondary to a rise in intraosseous pressure due to fatty infiltration following steroid therapy. While contributing factors from underlying disease processes confound this condition, elucidating the mechanism responsible for ON in patients using corticosteroids has been an area of intense research. 

Studies have revealed abnormalities of lipid metabolism in both humans and animals following exposure to corticosteroids [80, 103–106]. In animal studies, induced hypercortisolism resulted in adipocyte hypertrophy, hyperlipidemia, fatty liver, and systemic fat emboli. Although marrow edema and fatty necrosis was frequently observed, in quadrupedal studies, no area of bone necrosis or articular collapse could be identified. On the other hand, chickens treated with steroids did show evidence of ON, suggesting that differences in biomechanics between bipedal and quadrupedal species may be an important contributing factor [54]. 

Studies in mice and humans have shown that dexamethasone, given in a dose and time-dependent fashion, induces the differentiation of bone marrow derived stem cells into adipocytes, while inhibiting osteogenesis [53, 65, 71, 79]. Fat cell hypertrophy has been observed in histologic specimens of human femoral heads following treatment with dexamethasone for 5 days [62]. Dexamethasone has been shown to inhibit the expression of type-I collagen and osteocalcin, thereby suppressing the differentiation of bone marrow-derived stem cells into osteoblasts [53]. Prednisolone therapy has been found to decrease bone density and cancellous bone area while causing trabecular narrowing [107]. Mesenchymal stem cells derived from patients with corticosteroid-induced osteonecrosis of the femoral head have been shown to have lower proliferative ability, which may explain the low capacity for bone regeneration in these patients [68].

Lovastatin, when added to cell culture medium, had been found to inhibit adipogenesis and fat-specific gene expression caused by dexamethasone supplementation [54]. Additionally, lipid lowering agents counteract the inhibitory effect of steroids on osteoblastic gene expression [54]. These findings have been demonstrated both *in vitro* and *in vivo* [54, 74, 79]. In a supporting study, mesenchymal cells transplanted into host mice following transfection with a traceable gene demonstrated increased adipogenesis following systemic steroid treatment [108]. Based on these observations, it has been postulated that steroid-induced ON may be due to intraosseous hypertension from excessive marrow fat accumulation or a shift in the differentiation of marrow stem cells into adipocytes, resulting in a reduction in the pool of stem cells available for osteoblast production, ultimately leading to insufficient repair and remodeling of necrotic bone. 

**Figure 2:**
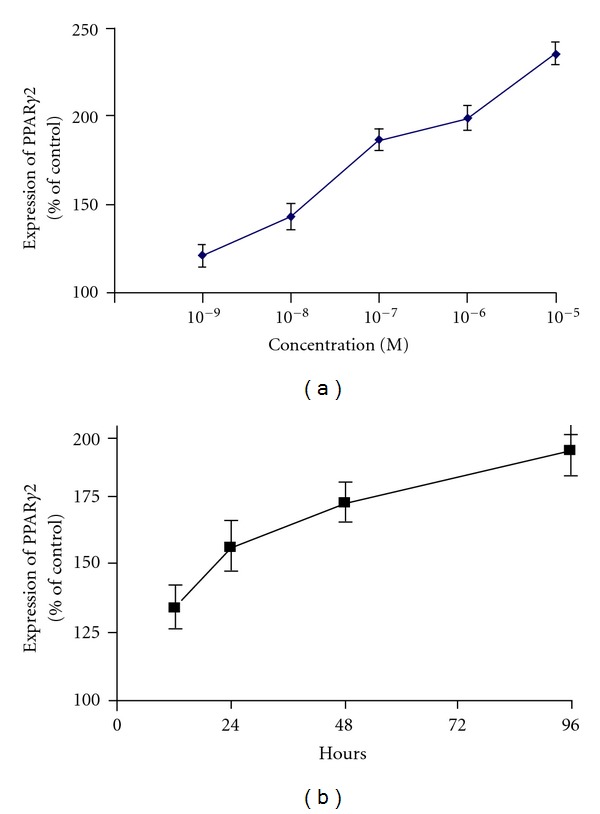
PPAR*γ*2 upregulation by dexamethasone is concentration and time dependent. Quantitation of the relative PPAR*γ*2 gene expression of the D1 cells treated with (a) different concentrations of dexamethasone for 48** **h and (b) 10^−7^ mol/L dexamethasone for different time points. Error bars represent the standard deviation of triplicate experiments (*P < 0.05). (Reprinted, with permission, from: [56].)

**Figure 3:**
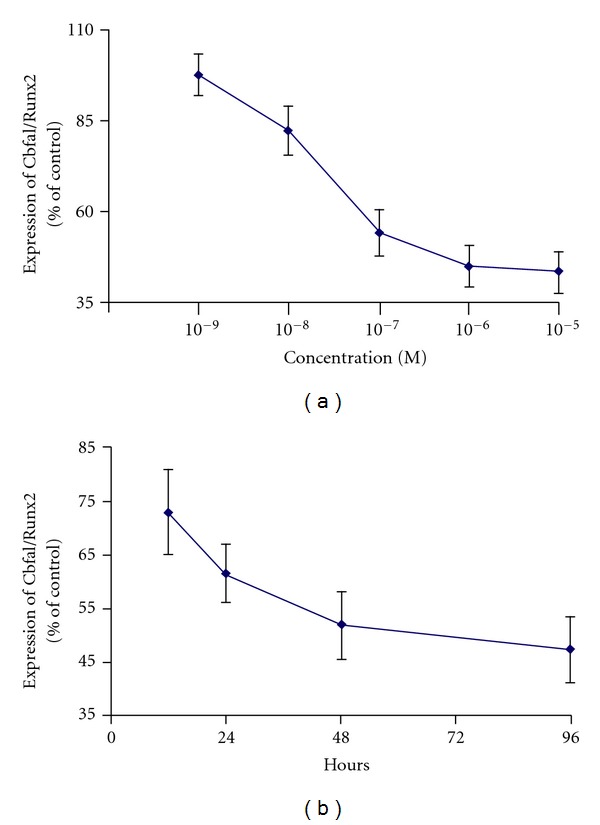
Concentration and time response of Cbfa1/Runx2 mRNA downregulation by dexamethasone. Quantitation of the relative Cbfa1/Runx2 gene expression of the D1 cells treated with (a) different concentrations of dexamethasone for 48 h and (b) 10^−7^ mol/L dexamethasone for different time points. Error bars represent the standard deviation of triplicate experiments (*P < 0.05). (Reprinted, with permission, from: [56].)

**Figure 4:**
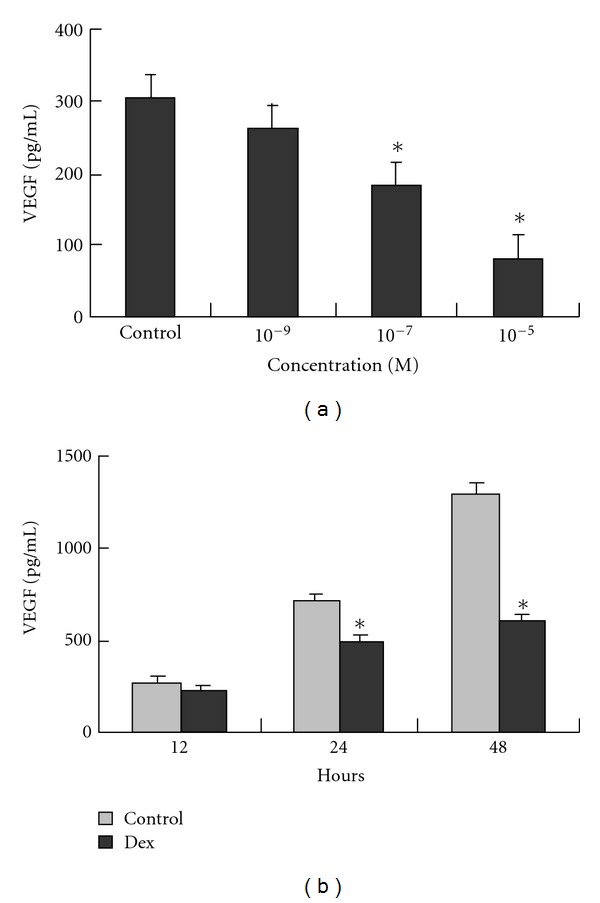
Inhibition of VEGF production of D1 cells by dexamethasone is dose and time dependent. VEGF expression in supernatant medium of the D1 cells treated with (a) different concentrations of dexamethasone for 24 h and (b) 10^−7^ mol/L dexamethasone for different time points was assessed by ELISA. Each column shows mean ± SD of data from three experiments (*P < 0.05). (Reprinted, with permission, from: [56].)

The mechanisms by which steroid-treated mesenchymal stem cells demonstrate increased adipogenesis and decreased osteogenesis have been studied at the molecular level [53, 55, 56]. Peroxisome proliferator activated receptor-*γ* (PPAR-*γ*) and core-binding factor a1 (Cbfa1) are transcription factors found to be important in the differentiation of pluripotent cells into adipogenic and osteogenic cell lines, respectively. Dexamethasone has been shown to increase mRNA expression of PPAR-*γ* (Figure 2) and decrease mRNA expression of Cbfa1 (Figure 3). These findings support the idea that dexamethasone promotes adipogenesis while inhibiting osteogenesis. Additionally, these studies also suggest that dexamethasone impairs angiogenesis by suppressing the production of VEGF (Figure 4). Osteoblasts derived from femoral heads have been found to exhibit downregulation of VEGF within 24 hours of incubation with glucocorticoids [67]. In a rabbit steroid-induced osteonecrosis model, however, VEGF levels increased to peak levels 3 days after methylprednisolone treatment [59]. These studies suggest that while steroids inhibit VEGF expression in isolated osteoblast cultures, steroid associated ischemic events occurring *in vivo *likely contribute to an upregulated VEGF response. The shunting of osteoprogenitor cells into the adipocytic pathway in conjunction with the suppression of angiogenic growth factor production may at least partially explain the basis of steroid-induced ON. 

Using a porcine model, Drescher et al. investigated the effects of methylprednisolone on the response of femoral head epiphyseal arteries to vasoactive substances [57]. Steroid-treated vessels demonstrated an increased response to endothelin-1 and a decreased response to bradykinin in comparison to untreated controls. Because endothelin-1 effectively vasoconstricts while bradykinin vasodilates, the overall conclusion of this work is that methylprednisolone, when coupled with vasoactive substances, modulates femoral head epiphyseal artery contraction. These findings support the hypothesis that the pathomechanism of steroid-induced femoral head ON is related to disturbed femoral head blood flow.

Ischemia and subsequent reperfusion of the femoral head is also thought to contribute to ON. Drescher et al. studied the influence of short-term high-dose steroid treatment on femoral head reperfusion following ischemic insult in a porcine model [109]. Femoral head ischemia was achieved by pressurizing the hip to 250 mm Hg for 6 hours. Radiolabeled microspheres released into the bloodstream were used to estimate blood flow to the femoral head. In comparison to untreated controls, methylprednisolone treatment was not shown to have an effect on reperfusion of the femoral head following an ischemic insult. Despite these findings, baseline blood flow was profoundly reduced in groups treated with methylprednisolone.

Zaidi et al. have suggested that adrenocorticotropic hormone (ACTH) may protect against methylprednisolone-induced osteonecrosis of the femoral head [9]. In preliminary studies, this group documented functional ACTH receptors on osteoblasts which, when activated, enhanced osteoblastic proliferation [110]. In an *in vivo *osteonecrosis model, rabbits treated with depomedrol plus ACTH for 1 month demonstrated fewer signs of trabecular necrosis and increased expression of VEGF in comparison to groups treated with the steroid depomedrol alone [9]. Though statistical significance was not reached, quantitative DXA and tetracycline labeling showed a trend towards greater femoral head density and subarticular bone integrity in groups treated with ACTH. These findings support further examination into the efficacy of ACTH in preventing steroid-induced osteonecrosis.

### 5.2. Alcohol

One study showed a clear increase in the risk of femoral head ON in individuals consuming greater than 400 mL of alcohol per week [111]. Mouse and rabbit* in vitro *studies investigating the effect of alcohol on bone marrow stromal cells demonstrate that alcohol induces the differentiation of marrow stromal cells into adipocytes in a dose dependent manner [10]. Alcohol-induced a significant increase in serum triglyceride and cholesterol levels, in addition to liver and bone marrow fatty infiltration. In the subchondral areas of the femoral head, fat cell hypertrophy and proliferation were observed. Triglyceride deposition in osteocytes lead to pyknosis and an increased percentage of empty osteocyte lacunae. None of these findings were apparent in untreated control groups. Alcohol treated groups demonstrated intracellular lipid deposition which ultimately lead to death of osteocytes. Cells treated with ethanol showed diminished alkaline phosphatase activity and expression of osteocalcin. Similar to the effects of corticosteroids, alcohol increases adipogenesis and decreases osteogenesis. Unlike the effect of steroids on stromal cells, alcohol-treated cells did not show increases in PPAR-*γ* expression, supporting the idea that alcohol influences fatty acid metabolism through a differing mechanism.

Wang et al. have suggested, from *in vitro *and* in vivo* studies, that the Chinese herbal puerarin, with its antioxidative and antithrombotic effects, can prevent alcohol-induced osteonecrosis [69]. Cells treated with ethanol for 21 days, and mice treated with ethanol for up to 10 months demonstrated a decrease in alcohol-induced adipogenic gene expression when treated simultaneously with puerarin. It is postulated that the inhibitive effects of puerarin on bone-marrow adipogenesis leads to diminished fat marrow changes and subsequent maintenance of osteogenic differentiation of marrow stem cells.

In a study by Suh et al., marrow was collected from the proximal femurs of 33 patients following hip replacement for either alcohol-induced osteonecrosis of the femoral head or femoral neck fractures [65]. After isolating the mesenchymal stem cells and expanding them in culture, cells obtained from the osteonecrotic hips showed diminished osteogenic differentiation as compared to cells taken from fractured hips. 

The critical dose of corticosteroids and alcohol necessary to induce ON is largely unknown. It appears that serum corticosteroid concentration is a more important risk factor than cumulative dose or duration of therapy. There exists a strong association between daily total dose and oral dosing (as opposed to parenteral dosing) of corticosteroids in patients with femoral head ON [112]. Most cases of femoral head ON occur after high-dose oral corticosteroid treatment for longer than 1 month, though rare cases have occurred after shorter treatment intervals [113]. Changes indicative of evolving femoral head ON have been appreciated on MRI within three months in patients treated with high-dose prednisolone for 4–8 weeks [114]. These changes occurred prior to symptomatic onset.

From these studies it seems clear that both steroids and alcohol promote adipogenesis at the expense of osteoblastic proliferation or function. Although the exact molecular mechanisms may differ between these two implicating substances, the consequences of increased marrow fat, impaired vascularity, and diminished reparative capability all contribute to the final pathway of cell death. The role of an underlying genetic predisposition in the development of ON in these patients has not been fully elucidated but could explain why some chronic users of steroids or alcohol fail to acquire the disease [29, 73, 92]. 

In summary, the exact pathogenesis of ON is still unknown. However, many new insights have emerged from research in the last decade (Table 1). Recent studies have demonstrated that both steroids and alcohol promote adipogenesis while inhibiting osteogenesis and angiogenesis, leading to osteonecrosis and osteoporosis. Genetic factors and heritable coagulopathy including hypofibrinolysis and thrombophilia may also play an important role in the development of ON. Statins and anticoagulation therapy have shown promise in amelioration of ON. Further investigation in this area is needed.

## Figures and Tables

**Table 1:** New developments on pathogenesis of nontraumatic osteonecrosis and its clinical significance.

New findings	Clinical significance
Steroids enhance adipogenesis and inhibit osteogenesis and angiogenesis by marrow stem cells [53, 54, 56]	Therapeutic modulating marrow stem cell Statins have shown therapeutic effects [54, 56, 74]
Alcohol stimulate adipogenesis and inhibit osteogenesis by marrow stem cells [40, 65]	Therapeutic modulating marrow stem cell
Heritable hypofibrinolysis and thrombophilia [32, 34, 40, 50, 72]	Anticoagulation therapy is effective [75]
Genetic associations	
Mutations in the COL2A1 gene [30]	Familial high risk group
Polymorphisms in alcohol metabolizing enzyme gene [29]	High risk subgroup
Polymorphisms in the multidrug resistance gene 1 [73]	Screening high risk patients [73]
